# Mesenchymal cystic hamartoma of the lung mimicking simple pulmonary aspergilloma: a case report

**DOI:** 10.1186/s44215-023-00047-0

**Published:** 2023-05-18

**Authors:** Machiko Nishii-Mitsuaki, Chiaki Nakazono, Satoru Okada, Kenji Kameyama, Yoji Urata, Masayoshi Inoue, Yasuo Ueshima

**Affiliations:** 1 Department of General Thoracic Surgery, Japanese Red Cross Kyoto Daiichi Hospital, 15-749 Honmachi, Higashiyama-Ku, Kyoto, 605-0981 Japan; 2 Division of Thoracic Surgery, Department of Surgery, Graduate School of Medical Science, Kyoto Prefectural University of Medicine, 465 Kajii-Cho, Kamigyo-Ku, Kyoto, 602-8566 Japan; 3 Department of Pathology, Japanese Red Cross Kyoto Daiichi Hospital, 15-749 Honmachi, Higashiyama-Ku, Kyoto, 605-0981 Japan

**Keywords:** Mesenchymal cystic hamartoma, Aspergilloma, Lung

## Abstract

**Background:**

Mesenchymal cystic hamartoma is a rare pulmonary neoplasm, which occasionally presents with severe symptoms such as pneumothorax or hemothorax, and shows favorable outcomes following surgical resection. It presents as solitary or multiple cystic lesions and requires differentiation from cystic malignancies, lymphangiomyomatosis, and cystic adenomatoid malformation.

**Case presentation:**

We encountered a 46-year-old woman with mesenchymal cystic hamartoma mimicking simple pulmonary aspergilloma on diagnostic imaging. Chest computed tomography showed a cystic lesion 1.6 cm in diameter, with an intracavitary nodule. Surgical resection proved neither fungus nor malignancy, and the final pathological diagnosis of mesenchymal cystic hamartoma was made.

**Conclusions:**

Mesenchymal cystic hamartoma might show aspergilloma-like features on diagnostic imaging, in addition to the differential diagnosis of lung cancer.

## Background

Mesenchymal cystic hamartoma (MCH) of the lung is a rare pulmonary neoplasm that has favorable outcomes following surgical resection, although it occasionally presents with severe symptoms such as pneumothorax and hemothorax. MCH is radiologically characterized by cyst formation when the tumor reaches a size of 1 cm or more [1], and presents solitary or multiple cystic lesions in the lung on computed tomography (CT) scanning. Preoperative diagnosis of MCH is reportedly difficult due to similar radiographic features as other cystic neoplasms, such as pleuropulmonary blastoma, pulmonary metastasis of endometrial stromal sarcoma, lymphangiomyomatosis (LAM), and cystic adenomatoid malformation (CAM) [2]. Herein, we present a case of MCH mimicking simple pulmonary aspergilloma (SPA) on diagnostic imaging.

### Case presentation

**Fig. 1:**
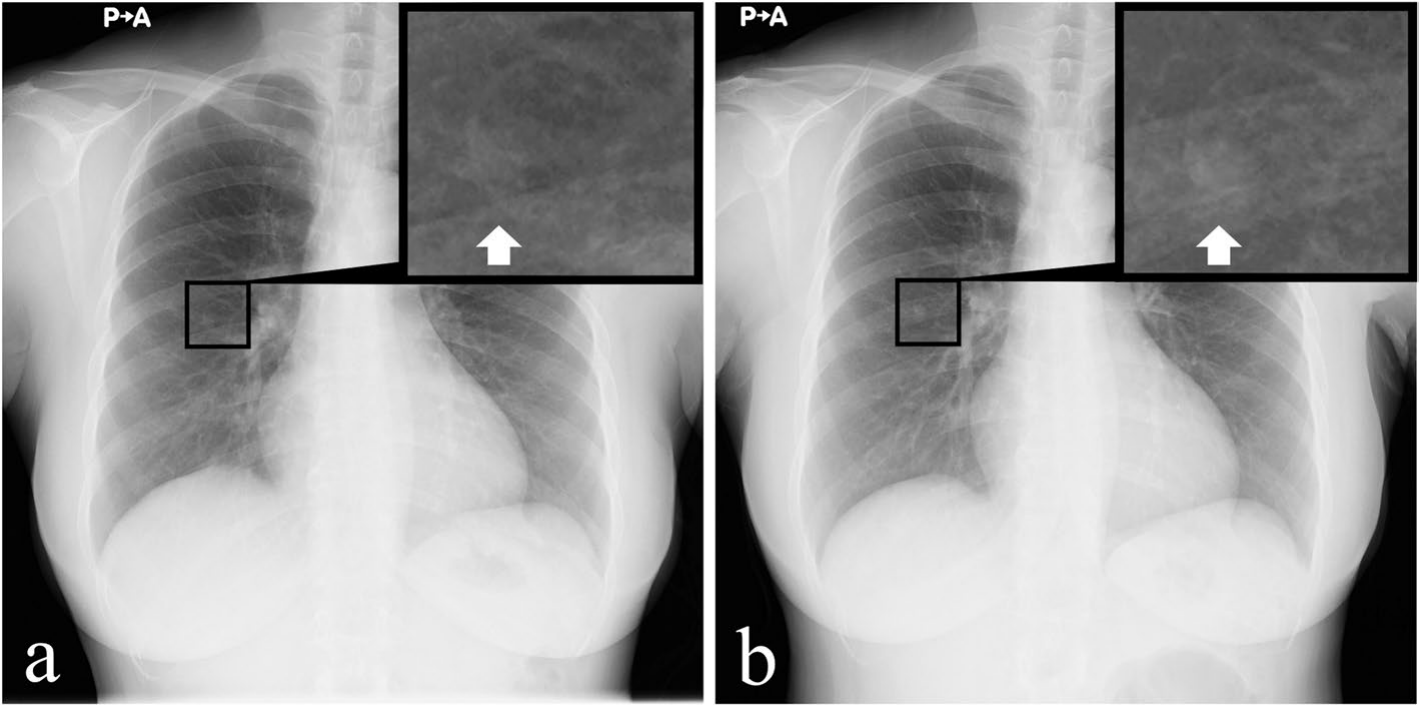
Chest X-ray findings: **a** A cyst with an intracavitary nodule was seen in the right middle lung field. **b** One year later, the intracavitary nodule had increased in size (enlarged view, arrowhead)

**Fig. 2:**
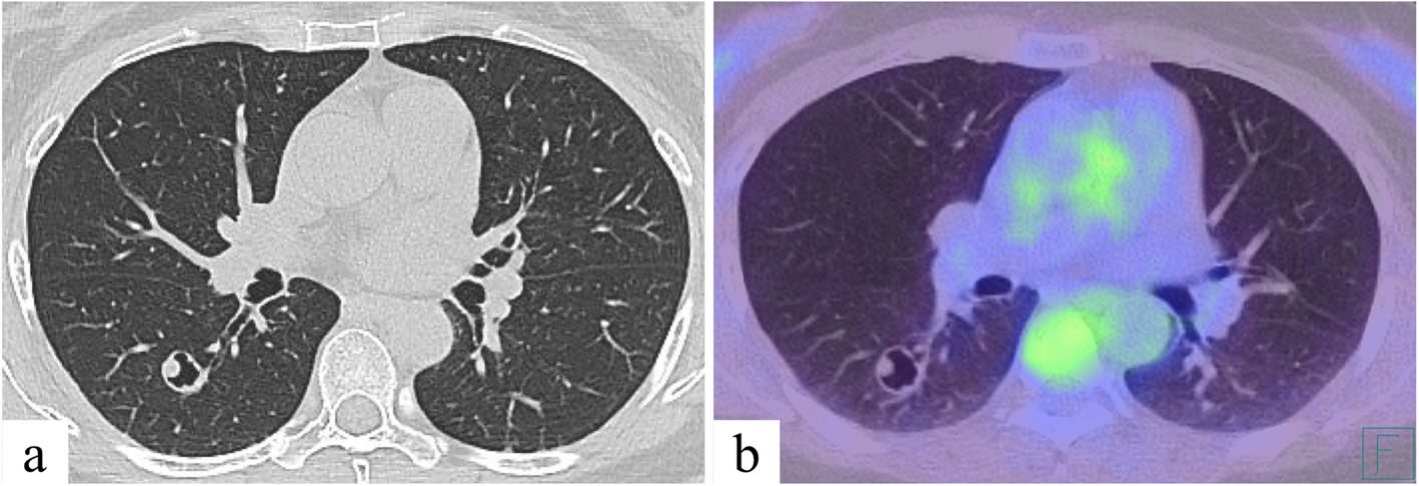
Preoperative imaging findings: **a** Chest computed tomography showed a thick-walled cystic lesion (measuring 1.6 × 1.5 cm) with an intracavitary nodule in the right lower lobe (S^6^). **b** No significant fluorodeoxyglucose accumulation was found in the cystic lesion in positron emission tomography/computed tomography

A 46-year-old woman who had a smoking history of 22 pack-years was referred to our hospital for thorough examination of an abnormal shadow on a chest X-ray taken during a physical examination. She had no symptoms, no allergies, was not on regular medications, and had no relevant medical history, including respiratory diseases. The chest X-ray revealed a tiny cystic lesion with an intracavitary nodule in the right middle lung field, which had enlarged over a period of one year, from her previous health examination (Fig. 1a, b). Chest CT showed a thick-walled cystic lesion (measuring 1.6 × 1.5 cm) with an intracavity nodule in the right lower lobe (S^6^) (Fig. 2a). Positron emission tomography/computed tomography (PET/CT) did not show any significant fluorodeoxyglucose (FDG) accumulation in the cystic lesion (Fig. 2b). Laboratory blood sample evaluation was negative for β-D glucan, while serum Aspergillus antigen was positive at a 0.7 cut-off index. The T-SPOT®-TB test and tumor markers (carcinoembryonic antigen, cytokeratin 19 fragment and pro-gastrin releasing peptide) were all negative. Based on these results, we suspected SPA as the preoperative diagnosis. Due to the risk of hemoptysis and the inability to rule out the possibility of lung cancer based on growth of the nodule, thoracoscopic right S^6^ segmentectomy was performed. Wedge resection was deemed difficult because the tumor was located far from the pleural surface. Intraoperative rapid pathological examination using a frozen section showed no evidence of malignancy. The surgical duration was 194 min and blood loss was minimal. She was discharged on postoperative day 6 after an uneventful clinical course.

**Fig. 3:**
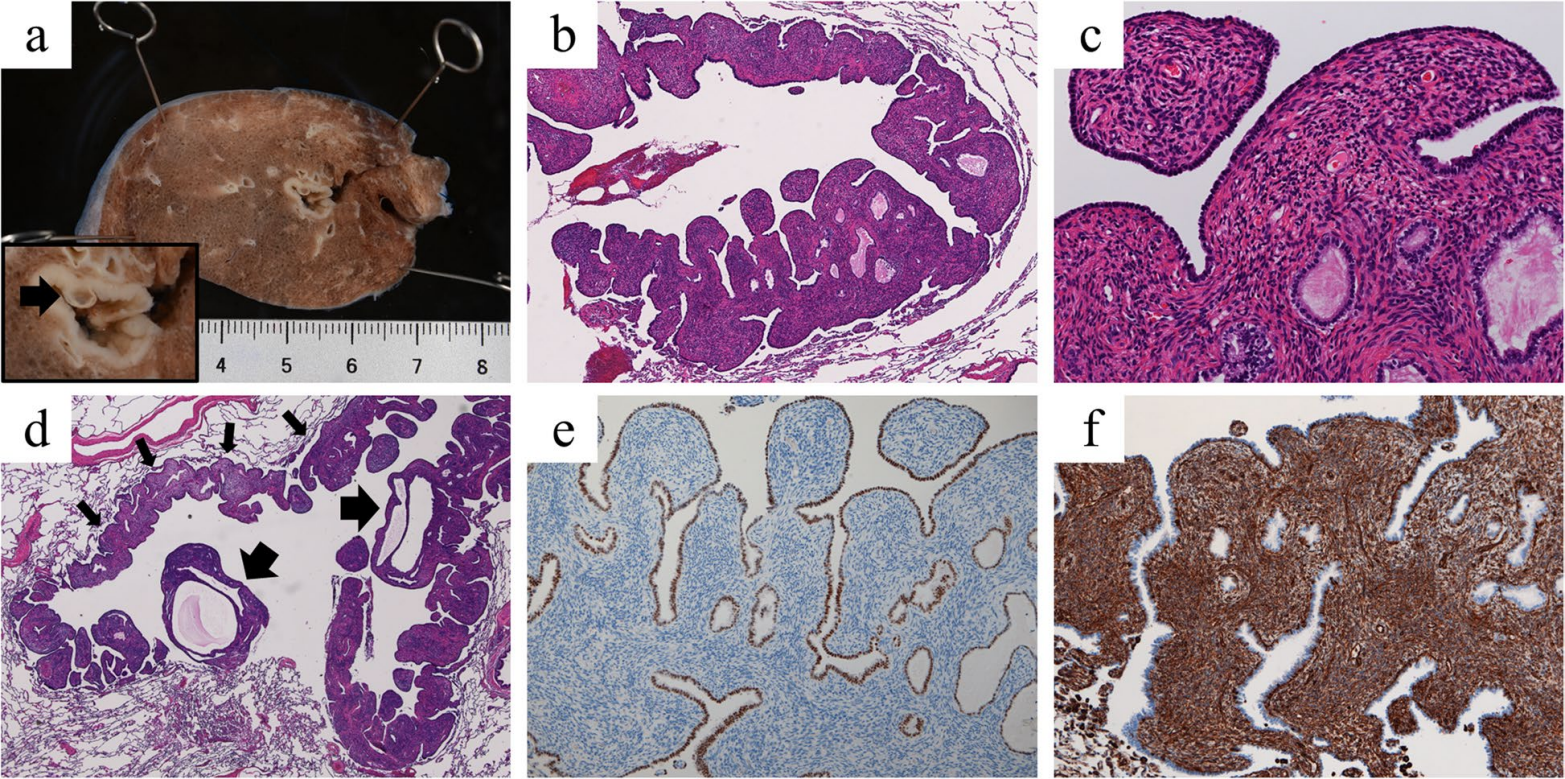
Postoperative pathology findings: **a** On gross examination, the cystic lesion (measuring 1.0 × 0.5 × 0.4 cm) had a thick wall, and the cyst wall partially protruded into the lumen (enlarged view, arrowhead). **b**, **c** Hematoxylin and eosin staining showed that the cyst wall was lined with monolayer columnar epithelium without atypia, and the stroma was composed mainly of short spindle cells along with some blood vessels and smooth muscle cells (B × 40, C × 200). **d** There were several microcysts within the cyst wall, some of which were dilated and protruded into the interior of the cyst (The cyst wall is indicated by small arrows and the dilated microcyst by large arrows). Some of these would have been visible as intracavitary nodules on CT (× 20). **e** The cells lining the cystic spaces were positive for TTF-1 (× 100). **f** The cells in the stroma were positive for vimentin (× 100)

Macroscopically, the resected specimen contained a thick-walled cystic tumor (measuring 1.0 × 0.5 × 0.4 cm) with the cyst wall partially protruding into the lumen (Fig. 3a). Microscopically, the lobulated cyst wall had a broad stroma and was lined with normal respiratory epithelium. The stroma was composed mainly of short spindle cells, which appeared to be mesenchymal cells, along with some blood vessels and smooth muscle cells (Fig. 3b, c). There was no cartilaginous component. Within the cyst wall were several microcysts whose surfaces were covered by respiratory epithelium, some of which were dilated and protruded into the cavity. Protrusion of the wall containing these dilated cysts into the interior would have been visible as the intracavitary nodules on CT (Fig. 3d). On immunohistochemical examination, the cells lining the cystic spaces were positive for TTF-1 and the cells in the stroma were positive for vimentin (Fig. 3e, f). The MIB-1 index was low (< 1%). No fungi were detected histologically. Since there was no histopathological evidence of malignancy and the lesion consisted of a single lesion in an adult female with no significant clinical symptoms, lung cancer and other cystic neoplasms, such as pleuropulmonary blastoma, pulmonary metastasis of endometrial stromal sarcoma, LAM, and CAM were ruled out. A final diagnosis of MCH was made.

The patient showed no recurrence for 18 months following the surgery, as seen on follow-up using CT scanning.

## Discussion

We experienced a case of MCH with a rare presentation mimicking aspergilloma. In the present case, SPA was initially suspected in addition to lung cancer, because of the CT finding of a single cystic lesion with wall thickening and an intracavitary nodule without FDG-uptake, along with elevation of serum Aspergillus antigen levels. A PubMed search for “mesenchymal cystic hamartoma, lung, from 1986 to 2022” revealed no previous reports of MCH requiring differentiation from Aspergillus infection, and, to the best our knowledge, the present case is the first such report.

Previous reports have described MCHs presenting as nodules or cysts, some with septae within the cyst, but with no specific CT findings [3]. SPA, on the other hand, is characterized by a cavitary lesion with crescent-shaped air around the fungus ball (crescent sign) on CT images. The intracavity fungus ball is sometimes mobile, and the lesion reportedly shows no increase in size over several months and typically presents no evidence of surrounding inflammation [4]. CT in the present case showed a single cystic lesion with mild wall thickening with poor inflammatory findings and an intracavitary nodule, with imaging findings similar to SPA, although mobility of the nodule was not evident. Although none of the previously reported MCH cases mentioned aspergillus infection in the differential diagnosis, a case of MCH presenting with cystic lesions and internal nodules was also reported by Leopizzi et al. [5]. MCH and SPA are similar in that they are both slow-growing tumors, which might make it difficult to distinguish between them on imaging. Although the result for Aspergillus antigen was a false-positive in this case, the cause was not determined. False-positive results have previously been reported to be caused by the use of β-lactam antibiotics and the ingestion of foods (grains, etc.) contaminated with galactomannan in the environment in patients with impaired intestinal mucosal barrier function, such as immunocompromised hosts [6]. In the present case, however, there was neither a history of antibiotic usage nor immunodeficiency.

MCH of the lung, which was first described in 1986 [1], is a rare tumor originating from primitive mesenchymal cells, and differs from ordinary hamartomas in that it forms cysts. MCH is diagnosed based on the clinical findings of slow growth and lack of symptoms relative to the size of the lesion, and by pathologic examination showing that the cyst wall is composed of primitive mesenchymal cells covered with normal respiratory epithelium [1]. Although the mechanism by which MCH, which initially develops as a nodule, forms a cavity when it reaches a size of about 1 cm has not been identified, previous reports have proposed the theory that cavitation occurs due to the destruction of bronchiolar and alveolar structures by proliferating cells, or the liquefaction of surrounding structures due to mucus secretion [1, 3]. In the present case, there was no obvious necrosis within the lesion, but there was mucus production within the microcyst that extended into the lumen, suggesting that mucus accumulated, expanded, and then drained out through the local bronchi and other pathways to form a cyst cavity. We consider that the cyst wall expanded, protruding into the lumen during its growth process, and increased while forming microcysts, which was detected on X-ray and CT over the course of one year. Since diagnostic biopsy of the cystic wall is unrealistic, there are no reports of a definitive preoperative diagnosis of MCH. In this case, diagnosis by bronchoscopic biopsy was considered to be difficult because of the small size of the lesion, which had a maximum diameter of 1.6 cm. Even if bronchoscopic bronchial biopsy had been performed, the treatment policy of surgery would have been unchanged regardless of the biopsy results, due to the observed growth in size of the lesion. While most cases are asymptomatic, there have been reports of pneumothorax, hemothorax, and, rarely, death due to hemoptysis in MCH cases [5, 7]. In addition, there have been reports of malignant transformation of these tumors, although only in infant cases [1, 8, 9]. Since the outcome is not always favorable, it might be reasonable to perform surgery with diagnostic and therapeutic intent. Additionally, we consider that careful postoperative follow-up is necessary in cases of MCH.

We experienced a case of MCH mimicking SPA. Although rare, MCH should be included in the differential diagnosis of cases of suspected SPA or lung cancer with cystic wall thickening on imaging.

## Data Availability

All data generated or analyzed during this study are included in this published article.

## Abbreviations

MCH: Mesenchymal cystic hamartoma
CT: Computed tomography
LAM: Lymphangiomyomatosis
CAM: Cystic adenomatoid malformation
SPA: Simple pulmonary aspergilloma
PET/CT: Positron emission tomography/computed tomography
FDG: Fluorodeoxyglucose

## References

[1] Mark EJ. Mesenchymal cystic hamartoma of the lung. N Engl J Med. 1986;315(20):1255–9. 3773938 10.1056/NEJM198611133152004

[2] Zhu H, Huang S, Zhou X. Mesenchymal cystic hamartoma of the lung. Ann Thorac Surg. 2012;93(6):e145–7. 22632532 10.1016/j.athoracsur.2011.12.041

[3] Yuan L, Wang S, Wei J, Yang K, Mao Y. Mesenchymal cystic hamartoma of the lung. Medicine. 2022;101(1):e28242. 35029876 10.1097/MD.0000000000028242 PMC8735712

[4] Denning DW, Cadranel J, Beigelman-Aubry C, Ader F, Chakrabarti A, Blot S, et al. Chronic pulmonary aspergillosis: rationale and clinical guidelines for diagnosis and management. Eur Respir J. 2016;47(1):45–68. 26699723 10.1183/13993003.00583-2015

[5] Leopizzi M, Cerbelli B, Merenda E, Pignataro MG, Bassi M, Venuta F, et al. Mesenchymal cystic hamartoma presenting with pneumothorax: case report and review of the literature. Gen Thorac Cardiovasc Surg. 2020;68:1573–8. 32361809 10.1007/s11748-020-01370-x

[6] Mennink-Kersten MASH, Donnelly JP, Verweij PE. Detection of circulating galactomannan for the diagnosis and management of invasive aspergillosis. Lancet Infect Dis. 2004;4(6):349–57. 15172343 10.1016/S1473-3099(04)01045-X

[7] Chadwick SL, Corrin B, Hansell DM, Geddes DM. Fatal haemorrhage from mesenchymal cystic hamartoma of the lung. Eur Respir J. 1995;8(12):2182–4. 8666115 10.1183/09031936.95.08122182

[8] Hedlund GL, Bisset GS, Bove KE. Malignant neoplasms arising in cystic hamartomas of the lung in childhood. Radiology. 1989;173(1):77–9. 2781034 10.1148/radiology.173.1.2781034

[9] Bove KE. Sarcoma arising in pulmonary mesenchymal cystic hamartoma. Pediatr Pathol. 1989;9(6):785–92. 2602233 10.3109/15513818909022388

